# Synthesizing Explainability Across Multiple ML Models for Structured Data

**DOI:** 10.3390/a18060368

**Published:** 2025-06-18

**Authors:** Emir Veledar, Lili Zhou, Omar Veledar, Hannah Gardener, Carolina M. Gutierrez, Jose G. Romano, Tatjana Rundek

**Affiliations:** 1 Department of Neurology, University of Miami Miller School of Medicine, 1120 NW 14th Street, Suite 1370, Miami, FL 33136, USA; 2 Beevadoo e.U., Pfeifferhofweg 3b, 8045 Graz, Austria

**Keywords:** explainable machine learning, feature-importance aggregation, ensemble interpretability, small-data settings, WISFC

## Abstract

Explainable Machine Learning (XML) in high-stakes domains demands reproducible methods to aggregate feature importance across multiple models applied to the same structured dataset. We propose the Weighted Importance Score and Frequency Count (WISFC) framework, which combines importance magnitude and consistency by aggregating ranked outputs from diverse explainers. WISFC assigns a weighted score to each feature based on its rank and frequency across model-explainer pairs, providing a robust ensemble feature-importance ranking. Unlike simple consensus voting or ranking heuristics that are insufficient for capturing complex relationships among different explainer outputs, WISFC offers a more principled approach to reconciling and aggregating this information. By aggregating many “weak signals” from brute-force modeling runs, WISFC can surface a stronger consensus on which variables matter most. The framework is designed to be reproducible and generalizable, capable of taking important outputs from any set of machine-learning models and producing an aggregated ranking highlighting consistently important features. This approach acknowledges that any single model is a simplification of complex, multidimensional phenomena; using multiple diverse models, each optimized from a different perspective, WISFC systematically captures different facets of the problem space to create a more structured and comprehensive view. As a consequence, this study offers a useful strategy for researchers and practitioners who seek innovative ways of exploring complex systems, not by discovering entirely new variables but by introducing a novel mindset for systematically combining multiple modeling perspectives.

## 1. Introduction

The pervasive integration of Artificial Intelligence (AI) and Machine Learning (ML) imposes increased interpretability and transparency, especially as these systems influence significant decisions [1–3]. The demand for explainable algorithms stems from diverse stakeholders, including affected individuals, regulators, domain experts, and developers, each with potentially different explanation requirements [4]. This concern is further echoed in safety-critical contexts, where AI introduces non-deterministic behavior that traditional engineering methods struggle to assure [5]. As a result, reliable and transparent interpretability techniques, such as structured methods for aggregating explanations, are essential to reinforce trust and validate behavior in complex, real-world systems. The lack of understanding surrounding machine-learning system decisions presents a disadvantage in various domains, notably in healthcare and autonomous systems [2]. Furthermore, new regulations and the requirements of highly regulated fields mandate the suitability and verifiability of algorithmic decisions [3]. Consequently, there is a growing demand for the ability to question, comprehend, and trust machine-learning systems, making interpretability indispensable.

Explainable AI (XAI) research aims to address the black box problem by developing methods to make AI decisions understandable [6]. The literature distinguishes between inherently interpretable models and complex models that require post hoc explanation techniques to reveal their inner workings [7]. While the latter often achieve superior accuracy, they rely on external methods to provide meaningful insights into their predictions [8]. Popular post hoc techniques include Local Interpretable Model-agnostic Explanations (LIME) [9] and SHapley Additive exPlanations (SHAP) [10], which aim to explain predictions by quantifying the contribution [8,11] or importance [1] of input features, respectively.

A significant challenge in XAI research and practice is the inconsistency and disagreement observed between explanations generated by different state-of-the-art methods for the same model prediction [12]. Practitioners frequently encounter these disagreements, often resorting to ad hoc heuristics or expressing uncertainty regarding how to resolve them reliably. This highlights a critical gap and the potential for practitioners to rely on misleading explanations [12]. Evaluating the quality of explanations is a critical but often missing step in XAI research, with a lack of consensus on standardized metrics and practices [6,13]. Evaluation approaches can include functionality-grounded (objective metrics), application-grounded (domain-specific tasks), and human-grounded (user studies) assessments [13].

We investigate the problem of explanation disagreement, particularly in the context of feature-importance ranking, and propose a methodology to derive more stable and reliable factor importance measures by aggregating explanations from multiple models. This research addresses two key questions: whether brute-force modeling using a large number of models can improve the reliability of identified predictors and whether aggregating explanations across those models serves as an effective mechanism to achieve that goal.

We present a cross-ensemble feature ranking methodology leveraging the WISFC procedure and the AGREE method [14]. The approach aggregates local explanations from an ensemble of diverse machine-learning models to produce a more robust aggregate feature-importance vector. The methodology is evaluated using several classification and regression datasets, including a non-objective-oriented healthcare dataset, demonstrating its ability to provide stable and reliable feature rankings compared to single explanation methods [1]. This is particularly valuable for obtaining dependable insights from challenging datasets, such as those that may be small and imbalanced. WISFC offers a more scientifically grounded approach to ensemble interpretability, enhancing confidence in predictor robustness and advancing the practice of explainable AI for structured data.

## 2. Background and Related Work

While rapid advancement in the efficacy of ML systems over the past decade has been notable, the adoption of increasingly complex models, particularly “black-box” techniques such as ensembles or Deep Neural Networks, has been impeded by their lack of inherent interpretability [14]. This challenge has propelled the field of XAI, which is now widely acknowledged as a crucial feature for the practical deployment of AI models and is becoming a prerequisite, even enforced by law in some regions [7]. XAI is also intertwined with the broader concept of Responsible Artificial Intelligence (RAI), which emphasizes principles such as fairness and accountability alongside model explainability [7]. A solid understanding of the underlying concepts, processes, and challenges of ML and Deep Learning (DL) is required for their effective implementation [15]. We review the existing literature on XAI methods and taxonomies, examine current practices, and highlight key challenges relevant to achieving reliable explanations from complex and potentially multiple ML models, addressing our research questions.

Research in XAI has introduced many methods, and several surveys now group these techniques using different criteria [4,6,7]. A primary distinction is often made between transparent or intrinsically interpretable models and post-hoc explainability techniques applied to opaque or black-box systems [4,7]. Taxonomies also categorize methods by the scope of interpretability (local, explaining individual predictions, versus global, explaining overall model behavior) [4] and dependency on the model (model-agnostic techniques applicable to any ML model versus model-specific techniques tailored to certain architectures) [4,7]. Popular post-hoc model-agnostic techniques include LIME and SHAP, which aim to provide insights into model predictions, often by estimating the importance or contribution of input features [7]. While a wide array of techniques exists, a standardized framework for evaluating the quality and effectiveness of the generated explanations is still missing [13]. Evaluation often involves quantitative metrics, user-focused assessments, or clinical validation in domain-specific applications [6,13].

Despite the advancements in XAI methodologies, significant challenges remain, particularly when applying and synthesizing explanations across multiple models or complex ensemble structures. One notable issue is the inconsistency or “disagreement problem” that can arise between explanations provided by different XAI methods or even the same method applied to slightly different models or datasets [13,14]. For instance, the factor contribution values and rankings provided by methods like Kernel SHAP can differ across various models for the same dataset [1]. This variability relates to the challenge of achieving stability of explanations, specifically the consistency of the set of features identified as important [11]. The differing importance ranks for factors across models highlight that a single explanation may not reliably reflect the true underlying importance. Addressing these inconsistencies and ensuring the stability and reliability of identified important features is crucial for fostering trust in AI systems [1]. The complexity of implementing taxonomies across diverse methods also presents a challenge [4]. The ability to synthesize information from potentially multiple models is relevant, especially considering if an ensemble approach can yield more reliable predictors than single models. Over time, the evolution of explainability has followed a clear progression: initially, most machine-learning models were treated as full black boxes. Gradually, distinctions emerged as some models offered intrinsic transparency. Next came ranking feature importance within individual models, followed by attempts to compare these rankings across models. Ultimately, approaches like WISFC advance this trajectory by aggregating these rankings into a multidimensional view that captures complementary signals from diverse models. In essence, each model simplifies the problem space from a particular perspective, and WISFC combines these perspectives into a more structured, comprehensive interpretation. Therefore, developing methods that can aggregate insights and provide stable, robust explanations across multiple ML models, particularly from structured datasets, represents a key area of research.

## 3. Proposed Method: Weighted Importance Score and FrequencyCount (WISFC)

We introduce WISFC, a method that combines feature-importance outputs from multiple explainable models by integrating two metrics: Weighted Importance Score (WIS) and Frequency Count (FC). WIS measures a feature’s average importance across models, while FC captures how often it appears among each model’s top-k. WISFC balances importance, magnitude, and stability, directly addressing our research questions by enabling reliable, reproducible aggregation of diverse explainers.

### 3.1. Overview and Rationale

WISFC addresses two central questions: “Can using many models yield a more reliable set of predictors?” and “How does WISFC improve the identification of stable predictors?” Instead of relying on visual comparisons of overlaps, WISFC quantifies two complementary metrics for each feature:

- WIS: its mean normalized importance (e.g., inverse-rank or scaled score) over all models
- FC: the proportion of models ranking it in their top-k.

By combining these, WISFC highlights features that are both strongly and consistently important. It is model-agnostic (i.e., any numeric importance output can be normalized and fed into WISFC) and particularly useful when data are scarce, trading brute-force modeling diversity for feature-selection robustness.

### 3.2. Aggregating Importance Scores (Weighted Importance Score)

The first component of *WISFC* is the Weighted Importance Score (WIS). To compute WIS, we proceed as follows. Suppose we have N different trained models or explanation outputs (these could be entirely different model types or the same model type trained with different hyperparameters or on different folds—WISFC works in either case). Each model j provides an importance value $I_{j}(f)$ for each feature f. This $I_{j}(f)$ could be, for example, the mean absolute SHAP value of feature f for model j, the weight coefficient magnitude in model j, or the feature’s permutation importance score—whatever is appropriate for that model. We do not assume all models use the same importance metric since tree-based models and linear models, for instance, have different scales; therefore, as a pre-processing step, we normalize or rank-transform each model’s importance outputs into a comparable scale [14]. A simple and robust choice is to convert each model’s raw importance scores into ranks (1 = most important feature for that model, 2 = second, etc.) [14]. Alternatively, we can normalize by the sum of importances so that each model contributes a relative importance percentage. Let $R_{j}(f)$ denote the normalized importance (rank or scaled score) of feature f according to model j. Next, we assign a weight $w_{j}$ to each model’s importance contribution. In the basic version of WISFC, we can set all weights equal ($w_{j}=1/N$ for j=1,…,N), which effectively means every model’s opinion counts equally—so WIS becomes a simple average importance over models [14]. This unweighted average already embodies an ensemble consensus: features that consistently have high $I_{j}(f)$ across models will receive a high average, whereas features that are high in one model but low in others become “diluted.” However, WISFC also allows for performance-based weighting. If we have reason to trust some models more (for example, if Model A had higher cross-validated accuracy than Model B), we can give Model A a larger weight. A principled choice for weights is to use a SoftMax on performance so that all weights are positive and sum to 1 [1]. For instance, one could set the model weights $w_{j}$ proportional to their predictive performance using a SoftMax function over model accuracies:

$$w_{j}=\frac{exp\left({perf}_{j}\right)}{\sum_{k=1}^{N}exp\left({perf}_{k}\right)},$$

so that a model with better accuracy (or $R^{2}$ in regression) attains an exponentially higher weight in the importance aggregation [1]. This mirrors an existing approach [1], which demonstrates that incorporating model accuracy in the importance ranking improved the stability of the results. One must be careful here to use a performance metric evaluated on a validation set or via cross-validation (not the training set) to avoid overweighting an overfit model.

Finally, the Weighted Importance Score for feature f is computed as:

$$WIS(f)=\sum_{j=1}^{N}w_{j}R_{j}(f),$$

i.e., a weighted sum (or average) of the feature’s importance across all models. If we used rank-based $R_{j}(f)$, then WIS(f) can be interpreted as (inversely) the average rank of feature f. A lower WIS means the feature tended to rank high (1st, 2nd) across models, while a higher WIS means the feature often ranked lower (20th, 30th, etc.). We may invert it if we want a “higher is better” convention. If we used normalized scores, WIS(f) might range between 0 and 1 (if using importance percentages) or some relative scale. The key is that WIS(f) aggregates the strength of feature f’s importance as seen by all models collectively. This fulfills part of RQ4 by providing a quantitative, reproducible way to combine different models’ feature rankings, as opposed to eyeballing or narratively comparing them. Notably, this approach is similar to methods used in ensemble feature selection studies, where simple mean score aggregation was found effective and robust [16]. It also aligns with the “rank averaging” baseline in explainer aggregation research [14], ensuring that our method stands on a solid, validated foundation in terms of aggregation strategy.

### 3.3. Measuring Consistency (Frequency Count)

Aggregating scores into WIS addresses how important, on average, a feature is, but it does not directly capture how many models concur in citing that feature. A feature could attain a high WIS if it had extreme importance in a few models, even if half the models ignored it. For greater transparency, WISFC, therefore, includes a second metric: Frequency Count (FC). The Frequency Count FC(f) is essentially the number of models (out of N) for which feature f was deemed important by some criterion. There are a few ways to define “deemed important”: one practical way is to set a rank threshold (e.g., was f in the top 10 important features for model j?) or a score threshold (e.g., did f’s importance exceed 0.01 in normalized importance for model j?). The threshold can be tuned based on how many features one expects to be important in the problem. By default, using top-k lists is convenient—for instance, FC(f) could count in how many models f appeared in the top-k ranking (say k=10). If FC(f)=N, that means every single model considered f to be among the most important features; if FC(f)=1, only one model found f important (and the rest did not rank it in top-k). We can also express this as a percentage:

$$FC\%\left(f\right)=\frac{FC\left(f\right)}{N}\times 100\%,$$

This frequency-based view aligns with the intuitive practice researchers follow of checking how often a feature appears across different methods. Indeed, one of the alignment metrics in explainer disagreement research is exactly the top-k feature overlap between explainers [14]. Our FC(f) generalizes that concept to N models: it effectively measures the overlap of f across all model lists. A high FC is a sign of consensus. In the context of stability, FC relates to the concept of selection stability—for example, stability selection procedures [17] select features that appear in a high fraction of bootstrapped lasso models. Similarly, in ensemble feature selection, the frequency of a feature being selected is a key indicator of robustness [18]. Our use of FC is grounded in these ideas. It provides an easy-to-interpret number: if FC(f)=8 out of 10 models, one can straightforwardly explain that “eight different models independently found this feature important”, which can be persuasive for domain experts looking for reliable signals.

It is worth noting that FC by itself could be gamed (e.g., a feature might appear barely at rank k in many models but never high). That is why WIS and FC are complementary. We suggest using FC with a reasonably high k (so that we are counting fairly strong evidence of importance) or even combining it with a secondary condition (like “feature is in top-k and has a positive impact on outcome” if considering sign). FC directly measures consistency: features consistently identified across models yield high FC, whereas those important to only one model receive low FC, highlighting potential model-specific effects.

### 3.4. Integrating WIS and FC for Feature Ranking

**Figure 1.**
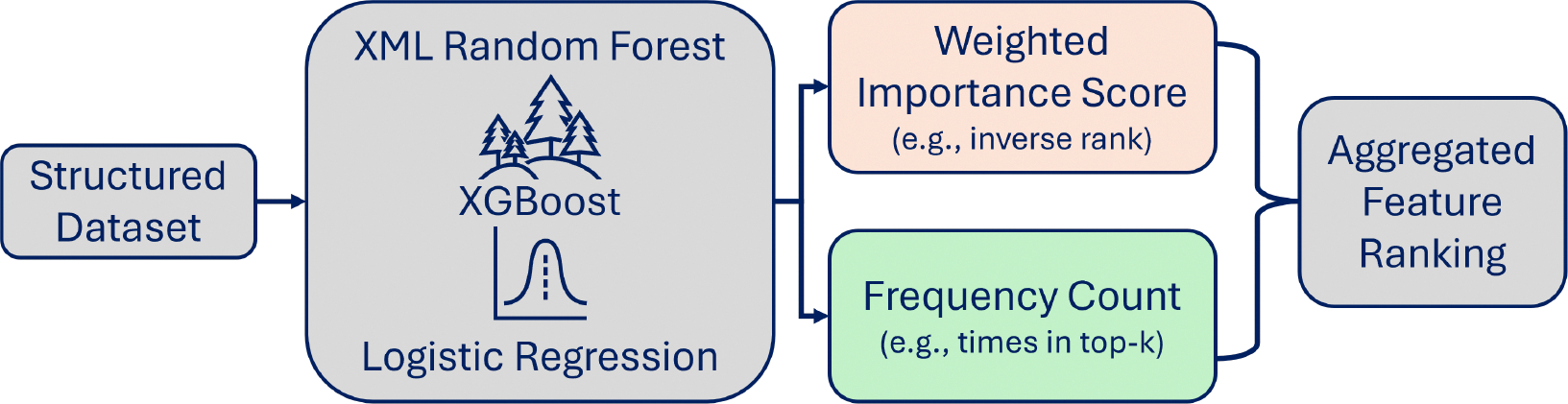
Simplified overview of WISFC method.

A simplified overview of WISFC is depicted in Figure 1. The figure illustrates how feature importance is extracted from multiple XML models, aggregated via Weighted Importance Score and Frequency Count, and combined into a final feature ranking.

Applying WISFC produces a feature table annotated with WIS and FC. To derive a final importance ranking or identify robust predictors, consider the following strategies:

- **Joint criteria:** We can set thresholds on both WIS and FC. For example, we might decide that a feature is robust if it has FC≥50% (appeared in at least half of the models) and is in the top quartile of WIS scores. This ensures we keep features that are not only frequent but also relatively highly ranked on average. Features meeting both criteria would be our high-confidence predictors. This approach is useful for binary decisions (selecting a subset of features for downstream use, e.g., in a final model or for reporting to stakeholders).
- **Composite score:** Alternatively, we could combine WIS and FC into a single composite importance metric. For instance, one might multiply a feature’s average importance by a factor related to its frequency (somewhat like a weighted scoring). A simple composite could be
$$C\left(f\right)=WIS\left(f\right)\frac{FC\left(f\right)}{N},$$
  effectively penalizing features that are not consistently found. However, interpreting this composite can be less intuitive, so often, it is clearer to keep WIS and FC separate and use them in tandem.
- **Ranking by WIS, tie-break by FC:** Another practical method is to primarily sort features by WIS (which gives an overall ranking from most important to least) and use FC as a secondary sort or a filter. For example, one can list features by descending WIS and then highlight those that have high FC as well (these would be the top stable features). Features with high WIS but low FC might be noted as “model-specific importance” and scrutinized further before trusting.

If we consider two features evaluated by five models:

Blood Pressure ranks: [1, 2, 4, 1, 3]
WIS \approx 2.2 (consistently high)
FC = 1.0 (top-3 in all models)
Cholesterol ranks: [1, 8, 2, 15, 1]
WIS \approx 5.4 (more variable)
FC = 0.8 (top-3 in four models)


A simple top-*k* overlap would treat both as majority-selected, but WISFC reveals that Blood Pressure never falls below fourth place and is thus more robust, whereas cholesterol’s low rank in one model lowers its consistency.

WISFC is model-agnostic and reproducible: any feature-importance output (e.g., SHAP, coefficients, permutation scores) can be normalized, scored, and aggregated. Researchers can replicate the procedure to verify WIS and FC values, ensuring transparent “consensus” selection. While extensions, such as tracking sign agreements across models, are straightforward, we focus on core rank-based aggregation. This framework directly addresses our research questions by enabling brute-force model synthesis and by offering a rigorous, quantitative alternative to ad hoc overlap methods. The next section applies WISFC to real data, illustrating its ability to surface stable predictors.

## 4. Case Study and Cross-Domain Examples

This section applies WISFC to a sample dataset and benchmarks it against rank-averaging and top-k overlap. We show that WISFC surfaces features that are both high-impact and stable across models. To demonstrate its generality, we include a non-medical example. This walkthrough also provides a clear, reusable template for deploying WISFC on any structured dataset.

### 4.1. Case Study: WISFC on a Small Clinical Dataset

To make the WISFC method more concrete, we present a case study drawn from our original research context (a small structured dataset in healthcare) [19]. The dataset in question involves, for example, a clinical cohort with a few hundred patients and a set of candidate predictors (demographics, lab tests, etc.) for a health outcome. Such datasets are often small due to the cost of data collection in medicine, and they pose a challenge for ML—many features might seem plausibly relevant, but with limited data, different models will latch onto different signals. In our case study, we applied a brute-force modeling strategy: we trained a diverse set of models, including a logistic regression, a decision tree, a random forest, a gradient boosting machine (XGBoost), and a support vector machine. We used repeated cross-validation to mitigate overfitting and obtained feature importance for each model using appropriate methods (coefficients for logistic, Gini importance for tree-based models, SHAP values for the SVM via a Kernel SHAP explainer, etc.). Each model thus produced a ranked list of 20 candidate predictors by importance.

The results were illuminating. Before applying WISFC, if we looked at each model separately, we saw considerable variation in their top predictors. For instance, Logistic Regression highlighted Age, Blood Pressure, and BMI as its top three; Random Forest gave the highest importance to Age as well, but also to Glucose Level and Cholesterol; XGBoost prioritized Glucose Level, Insulin, and Blood Pressure; the SVM (with SHAP) pointed to BMI, Age and a specific Genetic Marker as top features. The overlap between any two models’ top 3 was, at best, one or two features. A naive approach might have taken the intersection of all these sets—which would leave only Age and Blood Pressure as common to most. Indeed, those two features were the only ones that every model had ranked in its top five. Does that mean only Age and Blood Pressure are truly important? Common sense and domain knowledge suggest otherwise—Glucose and BMI, for example, are well-known risk factors in this medical context, and it was puzzling that not all models agreed on them. This is where WISFC helped uncover the nuance.

When we applied the Weighted Importance Score and Frequency Count aggregation, using equal weights for simplicity (each model 20% weight), we found that Age and Blood Pressure did indeed have high WIS and perfect FC (5/5 models). However, we also found Glucose Level had a high WIS—in fact, it was second only to Age in WIS—even though Glucose did not appear in the top 3 of the two models. Those two models still ranked Glucose moderately high (say, 4th and 6th), and the other three models ranked it very high (1st or 2nd). So its average rank was very good, and it had FC=4/580%). BMI similarly had FC=4/5 and a solid WIS (within the top 5 overall). On the other hand, the Genetic Marker that the SVM found important had FC=1/5(20%)—no other model thought it was important—and consequently, its WIS was low despite one strong vote. WISFC thus produced a ranked list of features by WIS, alongside their FC values, which we can summarize: e.g., Age (WIS1.4, FC100%), Glucose (2.2, 80%), Blood Pressure (3.0, 100%), BMI (4.0, 80%), cholesterol (5.6, 60%), Insulin (7, 40%), etc., down to features that never appeared in any top-10 (FC0%).

Comparing this WISFC-derived list to what we know from medical literature, we found it much more aligned with established risk factors. Age and Blood Pressure were expected, but WISFC rightly kept Glucose, BMI, and Cholesterol in the important set, whereas a simplistic intersection of top features might have wrongly discarded some of those due to disagreement between models. In other words, WISFC was able to extract a richer set of predictive variables than the strict common intersection while still being selective enough to ignore one-off spurious features. This directly supports our claim that brute-force modeling, in addition to structured aggregation, can overcome small-data limitations to yield a more comprehensive and reliable predictor list. By pooling evidence across models, we essentially increased the “effective evidence” for features: even if each model alone might overlook something, as long as several models found it important, WISFC rescued it with a high overall score. Notably, in this case study, we also compared the stability of the selected features via WISFC versus single-model selection. We observed that if we had just chosen the top 5 features from, say, the best single model (by accuracy), that set was quite sensitive to training data splits—different cross-validation folds yielded different top-5s. In contrast, the WISFC top features were far more stable across resampling; once multiple models were aggregated, the resulting importance ranking remained consistent in repeated experiments. This reinforces that aggregating multiple models’ explanations yields more robust results, echoing findings from other domains that ensemble feature selection improves stability by up to 30–40% [18].

### 4.2. Cross-Domain Example: Customer Churn Prediction

To illustrate WISFC’s versatility beyond healthcare, we present a hypothetical churn-prediction scenario, no real data were used. Imagine a customer dataset with features such as tenure, number of products, account balance, credit score, age, and online service usage labeled for churn. A data scientist might train three models: logistic regression, decision tree, and XGBoost. Each of those produces its own feature-importance ranking (e.g., standardized coefficients, Gini importances, SHAP values). While all three may concur that tenure belongs in the top ranks, they could disagree on other predictors such as credit score, number of products, or age. Applying WISFC follows the same workflow:

1. **Collect importance outputs** from each model.
2. **Normalize** and (optionally) **weight** them by validation performance (e.g., slightly favoring XGBoost if it achieved the highest AUC).
3. **Compute WIS** (average normalized rank) and **FC** (fraction of models ranking each feature in their top-k).

A plausible WISFC outcome might rank tenure highest (WIS≈1.3, FC=3/3), followed by the number of products (WIS≈2.1, FC=2/3) and credit score (WIS≈2.4, FC=2/3). Age may appear lower (WIS≈3.0, FC=1/3), reflecting its sole prominence in XGBoost. Compared with naive top-k overlaps or single-model explanations, WISFC would deliver a richer, more stable set of churn predictors to guide business analysts toward the most reliable factors for customer retention strategies. This demonstration confirms that WISFC applies to any structured-data domain where multiple models and interpretability are required.

WISFC yields a broader, more reliable feature set than rigid overlap rules while guarding against model-specific anomalies. Uncorroborated features (such as a lone genetic marker in the medical example or age in the churn scenario) remain visible but receive low FC scores as a cautionary signal. Conversely, predictors that never top any single model yet rank consistently high across several (e.g., BMI or credit score) are promoted by WISFC as robust candidates. This dual emphasis on consensus and magnitude provides the balanced strategy required for dependable knowledge discovery from small or noisy datasets.

## 5. Discussion

Traditional methods for merging multiple model explanations, such as comparing top-k overlaps or defaulting to a single model’s output, often rely on informal judgments and can produce inconsistent or misleading results. WISFC overcomes these limitations by introducing two reproducible metrics: Weighted Importance Score, which quantifies a feature’s average strength, and Frequency Count, which measures its consistency across models. Together, these metrics provide clear, numeric justification for why certain features are deemed robust.

Applying WISFC to small, noisy datasets demonstrates that training a diverse ensemble of models uncovers a broader set of plausible predictors than any individual model alone. Features that appear only sporadically, like a genetic marker flagged by one algorithm, are retained but receive a low-frequency score, signaling caution. Conversely, predictors that consistently rank well, even if never top, are elevated as reliable signals. This ensemble-driven approach rescues true positives (for example, BMI or cholesterol in our clinical case) that might otherwise be overlooked. Several key benefits emerge:

- Rigor and Reproducibility: Clear WIS and FC values replace subjective overlap heuristics, enabling others to replicate the aggregation and even apply statistical tests to these metrics.
- Domain-Agnostic Flexibility: Any model type and importance measure can feed into WISFC, making it equally suited to healthcare, marketing analytics, or engineering diagnostics.
- Robustness to Noise: By combining magnitude and consensus, WISFC down-weights one-off, model-specific quirks, and highlights features likely to generalize beyond the training data.
- Stable Conclusions: Minor changes, such as retraining one model, have a limited impact on high-frequency features, avoiding brittle exclusions inherent in strict intersection rules.

In sum, WISFC delivers a balanced, transparent framework for reliable knowledge discovery from structured data using multiple models.

WISFC excels at combining diverse model insights into a single, transparent feature ranking, but its effectiveness depends on the underlying models and data quality. When the ensemble consists of only minor variations of the same algorithm (e.g., different random seeds), WISFC will boost stability, as expected from any ensemble, but add little new information beyond conventional uncertainty intervals. To maximize its value, include genuinely diverse model types or architectures (linear vs. non-linear, tree-based vs. kernel methods, etc.).

WISFC also faithfully aggregates whatever biases are present. If all models inherit the same data skew or flawed feature engineering, a spurious signal may receive high WIS and FC. Rigorous data preparation, thoughtful feature selection, and domain-expert sanity checks remain essential.

Highly correlated or redundant predictors present another challenge. Different models may alternate between correlated features X and Y, resulting in moderate WIS and reduced FC for both, even though the underlying signal is strong. In such cases, interpreters should recognize feature groups rather than isolated variables. Clustering correlated features before aggregation is one extension, but at minimum, analysts should consider collective importance when interpreting WISFC rankings.

While correlated predictors may distribute importance across multiple features, we emphasize that, unlike traditional regression models where multicollinearity directly compromises coefficient estimates, ML models are primarily designed for prediction rather than strict causal inference. However, predictive modeling can still serve as a valuable basis for explanation. Prediction and explanation are not opposing paradigms but can complement each other [20], by capturing different aspects of the data-generating process. Thus, even when correlated variables share predictive capacity, their collective inclusion in WISFC contributes to a more comprehensive feature list that remains actionable for explanatory and decision-support purposes. Aggregating importance across models allows such features, whether independent or partially redundant, to be flagged for further clinical or domain-specific evaluation, which is often the practical objective in many structured-data applications.

Computationally, WISFC adds negligible overhead: the primary cost lies in training multiple models. For small to medium datasets, brute-force ensembles are practical, and deriving WIS and FC from existing importance lists is trivial. Even on larger datasets, practitioners can limit the ensemble to key model variants or use cross-validation folds as proxies. In all cases, the modest computational investment yields far greater clarity and robustness in feature interpretation.

For **practitioners** and **researchers** looking to apply WISFC, here are a few **guidelines and best practices**:

- **Assemble a diverse ensemble**. Include both simple (e.g., linear) and complex (e.g., tree-based, gradient boosting) models. Ensure each model is well-tuned so that importance differences reflect genuine explanatory variance, not poor fit.
- **Normalize importances**. Convert all feature-importance outputs to a common scale; rank-transformations are robust. Even SHAP values, which share units, can benefit from normalization to prevent scale discrepancies.
- **Select a sensible top-k for FC**. Choose k to reflect the expected number of relevant features (e.g., k = 10 for ten predictors). Avoid k so small that valid features hover just outside the cutoff or so large that FC loses discrimination. Test multiple k values; true robust features maintain high FC across reasonable ranges.
- **Apply performance-based weighting judiciously**. If one model significantly outperforms others on validation metrics, weight its importances more heavily. If model performances are comparable, use equal weights to avoid overemphasis. Always base weights on held-out or cross-validated scores to guard against overfitting.
- **Interpret WIS and FC in context**.
  - **High WIS and high FC**: robust predictors—report with confidence.
  - **High WIS and low FC**: model-specific signals—investigate further.
  - **Low WIS and high FC**: consistently moderate signals—consider for theory or follow-up testing.

By following these steps, practitioners can leverage WISFC to derive reliable, transparent insights from any structured dataset.

The introduction of WISFC carries some broader implications for explainable AI research and practice. It encourages a mindset shift from focusing on one model, one explanation, to multiple models, one synthesized explanation. This resonates with the general ensemble philosophy in ML—just as ensemble predictions are more reliable, ensemble explanations can be more trustworthy. It may prompt tool builders to incorporate features for explanation aggregation (e.g., an AutoML system could produce not just an ensemble model but an ensemble feature-importance report using something like WISFC). For high-stakes decision-making, using WISFC could become a best practice—for instance, regulatory guidelines on ML in healthcare might require not only model performance metrics but also some assessment of explanation robustness, as already suggested in early intelligible ML work in healthcare [21]; WISFC could serve as part of that assessment, signaling that certain conclusions are not model-dependent. The method also opens up several research avenues. One could extend WISFC to local explanations (aggregating explanations for individual predictions across multiple models to see if they agree on why a specific prediction was made—somewhat like AGREE’s approach [14], but focusing on agreement for a given instance rather than global features). Another extension is incorporating domain knowledge as an additional weight—for example, if prior knowledge suggests feature X is important, one might weight it slightly more in aggregation (though that veers into subjective territory, it could be useful in expert-in-the-loop settings). Additionally, evaluating WISFC on a wide range of datasets (small and large, different domains) empirically would help quantify how much it improves stability or predictive insight versus simpler methods; our work so far and related studies [16,18] suggest significant gains, but a systematic benchmark would solidify this.

## 6. Conclusions

We tackle the under-addressed challenge of combining multiple explainable model outputs on the same dataset. Today, practitioners routinely train diverse models and extract feature-importance scores. However, aggregation is often performed ad hoc (e.g., eyeballing top-k overlaps), inviting inconsistency and bias. To address this, we introduce Weighted Importance Score and Frequency Count (WISFC), a simple, reproducible framework that numerically fuses two dimensions:

1. Magnitude (WIS): the average, optionally performance-weighted rank of each feature across models.
2. Consistency (FC): the proportion of models ranking each feature within the top-k.

By jointly scoring features on impact and support, WISFC surfaces predictors that are both influential and robust. In a clinical case study, it recovered known risk factors (e.g., glucose, BMI) that single models might miss and demoted spurious, model-specific signals. Unlike informal overlap checks, WISFC provides clear thresholds (e.g., “90% of models and a top-5% average rank”) for asserting feature reliability.

Implementation is straightforward and broadly applicable: any set of importance measures (coefficients, Gini, SHAP) can be normalized, weighted, and aggregated. Whether in bioinformatics (ensuring gene-importance findings hold across algorithms) or finance (identifying risk factors that persist across models), WISFC enhances trust by anchoring explanations in consensus rather than chance.

As explainable AI gains traction in high-stakes domains, robust aggregation tools are essential. WISFC represents a step toward ensemble interpretability. Just as ensembles boost predictive accuracy, they can also stabilize explanations. Future work might integrate WISFC with interaction- or counterfactual-based explainers or develop statistical confidence bounds for its scores, further strengthening its rigor.

Ultimately, the core message remains: consistency breeds trust. By prioritizing features agreed upon by multiple models, WISFC transforms disparate interpretations into a cohesive, reliable narrative. We encourage its adoption in any setting where multiple models and limited data demand a reproducible, scientifically grounded approach to feature-importance aggregation.

## Data Availability

Restrictions apply to the availability of these data. Data were obtained from Hospitals participating in the Florida Stroke Registry and are available on request from Transitions of Care Stroke Disparities Study (TCSD-S) and the Florida Stroke Registry (FSR) https://floridastrokecollaboration.org/ (accessed on 15 June 2025) with the permission of respective hospitals.

## Abbreviations

XML: Explainable Machine Learning
WISFC: Weighted Importance Score and Frequency Count
AI: Artificial Intelligence
ML: Machine Learning
XAI: Explainable Artificial Intelligence
LIME: Local Interpretable Model-agnostic Explanations
SHAP: SHapley Additive exPlanations
RAI: Responsible Artificial Intelligence
DL: Deep Learning
WIS: Weighted Importance Score
FC: Frequency Count
